# Impact of Hypoxia over Human Viral Infections and Key Cellular Processes

**DOI:** 10.3390/ijms22157954

**Published:** 2021-07-26

**Authors:** Antonia Reyes, Luisa F. Duarte, Mónica A. Farías, Eduardo Tognarelli, Alexis M. Kalergis, Susan M. Bueno, Pablo A. González

**Affiliations:** 1 Millennium Institute on Immunology and Immunotherapy, Departamento de Genética Molecular y Microbiología, Facultad de Ciencias Biológicas, Pontificia Universidad Católica de Chile, Av. Portugal 49, Santiago E-8330025, Chile; azreyes@uc.cl (A.R.); lfduarte@uc.cl (L.F.D.); mrfarias@uc.cl (M.A.F.); eitognar@uc.cl (E.T.); akalergis@bio.puc.cl (A.M.K.); sbueno@bio.puc.cl (S.M.B.); 2 Departamento de Endocrinología, Facultad de Medicina, Escuela de Medicina, Pontificia Universidad Católica de Chile, Santiago E-8330025, Chile

**Keywords:** hypoxia, cellular response, viral response, DNA viruses, RNA viruses

## Abstract

Oxygen is essential for aerobic cells, and thus its sensing is critical for the optimal maintenance of vital cellular and tissue processes such as metabolism, pH homeostasis, and angiogenesis, among others. Hypoxia-inducible factors (HIFs) play central roles in oxygen sensing. Under hypoxic conditions, the α subunit of HIFs is stabilized and forms active heterodimers that translocate to the nucleus and regulate the expression of important sets of genes. This process, in turn, will induce several physiological changes intended to adapt to these new and adverse conditions. Over the last decades, numerous studies have reported a close relationship between viral infections and hypoxia. Interestingly, this relation is somewhat bidirectional, with some viruses inducing a hypoxic response to promote their replication, while others inhibit hypoxic cellular responses. Here, we review and discuss the cellular responses to hypoxia and discuss how HIFs can promote a wide range of physiological and transcriptional changes in the cell that modulate numerous human viral infections.

## 1. Introduction

Hypoxia is described as a reduction in the normal levels of oxygen due to a decreased availability or delivery of this gas to cells and tissues [1]. Under low oxygen levels, such as below 6%, or 40 mmHg of partial pressure (pO_2_) or less, tissues and cells can initiate a hypoxic response to adapt to this new condition [1]. Importantly, hypoxic conditions occur in several diseases, such as ischemic heart disease, ischemic stroke, and solid tumors, among others [2,3]. Under these low oxygen situations, numerous physiological changes will occur in tissues and cells, in such a way to rapidly adapt to this adverse condition. For instance, an increase in the respiration rate and its depth will occur at a whole organism level. At a cellular level, metabolic switches will take place, shifting from aerobic to anaerobic enzymatic pathways, altogether followed by a significant decrease in energy generation, which can be accompanied by the induction of a wide arrange of genes [1,2,4,5].

Hypoxia-inducible factors (HIFs) are transcriptional regulators that modulate the expression of hypoxia-dependent genes [1,2,4,5]. HIF-1 was first described in 1992 in human hepatocellular carcinoma cells (Hep3B) and immediately characterized as a critical regulator of oxygen tension levels [6]. This transcription factor was later found to belong to the PER-ARNT-SIM (PAS) family and was then characterized as a heterodimeric DNA-binding protein complex composed of a constitutively expressed β subunit and an oxygen-dependent α subunit [7,8]. Three isoforms of the α subunit have been described since then, namely HIF-1α, HIF-2α, and HIF-3α, giving rise to three different proteins HIF-1, HIF-2, and HIF-3, respectively [9,10]. When the α and β subunits dimerize, the HIFα/β heterodimer becomes transcriptionally active, translocates to the nucleus, and binds to hypoxia response elements (HREs), which are DNA consensus sequences that are present in the regulatory regions of HIF-target genes [1,7,8]. It is important to note that for this to occur, the α subunit of HIF must be stabilized, which happens under hypoxic conditions. In this scenario, degradation of the α subunit of HIF is inhibited thanks to the obstruction of the von Hippel-Lindau-containing (pVHL-containing) ubiquitin E3 ligase complex function, which otherwise targets the α subunit for degradation [7,8]. Alternatively, inhibition of the factor inhibiting HIF-1 (FIH-1) can also stabilize HIF. Under normoxic conditions (normal oxygen conditions), FIH-1, which is an asparaginyl hydroxylase, hydroxylates an asparagine residue in the C-terminal transactivation domain (C-TAD), which prevents the recruitment of transcriptional coactivators that bind to this region within HIF-1α. Notably, this process inhibits HIF-1α association with the transcriptional coactivator CBP/p300 and hence, the transcriptional activation of HIF-1α [7,8]. Importantly, prolyl-hydroxylase domain proteins (PHDs) hydroxylate two proline residues of HIF-1α (P^402^ and P^564^) [7,8]. Once these residues are converted to hydroxyproline, pVHL can bind to HIF-1α, leading to its degradation [7,8]. PHDs require oxygen as a co-substrate, and thus under hypoxic conditions, their enzymatic activity is inhibited, leading to HIF-1α stabilization [11].

Importantly, oxygen-independent regulation of HIF-1α also occurs. For instance, the activation of phosphatidylinositol 3-kinase (PI3K) increases HIF-1α stabilization [12]. This is mediated by an increase in HIF-1α protein translation through PI3K-target proteins, protein kinase B (Akt), and the downstream component mammalian target of rapamycin (mTOR) [12]. Noteworthy, the latter disrupts the integrity of the eukaryotic translation initiation factor binding protein (4E-BP1) via phosphorylation of the eukaryotic translation initiation factor 4E (eIF-4E) [13]. This modification inhibits cap-dependent mRNA translation and results in enhanced HIF-1α protein synthesis [13]. Additionally, mTOR can induce the phosphorylation of p70S6 kinase (S6K), which also promotes HIF-1α protein synthesis [13]. Moreover, some growth factors such as insulin-like growth factor (IGF-1) and epidermal growth factor (EGF) activate the tyrosine kinases receptor, which in turn activates PI3K, Akt, and the FKBP-rapamycin-associated protein (FRAP) [14]. Subsequently, FRAP induces the expression of HIF-1α in normoxic conditions [14]. RAS (rat sarcoma) protein can also be activated by growth factors, and therefore under these conditions, the RAS/RAF/MEK/ERK kinase cascade is stimulated [12]. Extracellular signal-regulated kinase (ERK) phosphorylates 4E-BP1, S6K, and MAP kinase-interacting kinase (MNK), which can also directly phosphorylate the eIF-4E [12]. ERK is also known to phosphorylate CBP/p300. As a result of all these signaling events, mRNA translation of HIF-1α and its transcriptional activity is increased [12]. Additionally, HIF-1α has been shown to be directly phosphorylated by p44/p42 mitogen-activated protein kinases (MAPK) (ERK1/2) in vitro and in vivo. Furthermore, two HIF-1α serine residues, S641 and S643, were detected by mass spectroscopy analyses with in vitro phosphorylated recombinant HIF-1α as ERK1/2 targets. Moreover, inhibition of this phosphorylation impaired nuclear accumulation and activity of HIF-1α [13,15]. Recently, it has been reported that the proviral integration site of the Moloney murine leukemia virus 1 (PIM1) kinase is able to directly phosphorylate HIF-1α at threonine 455 and HIF-2α at serine 435. These phosphorylations inhibit the ability of PHDs to bind and hydroxylate HIF-1α, thus promoting protein stabilization of these transcription factors [16].

Interestingly, the glycogen synthase kinase-3 isoform β (GSK3β) can directly phosphorylate HIF-1α and was found to directly phosphorylate HIF-1α in the oxygen-dependent degradation domain (ODDD), and the N-terminal transactivation domain (N-TAD) at different serine and threonine residues, such as S551, T555, S589, T498, S502, S505, T506, and S510. GSK phosphorylation of HIF-1α leads to its degradation through the multi-protein E3 ubiquitin ligase Skp, Cullin, F-box containing complex (SCF complex) [13]. Moreover, the lysine methyltransferase SETD7 (SET7/9) methylates the HIF-1α lysine residue at position 32 to induce protein destabilization and promote its proteasomal degradation [17]. Similarly, deacetylation of HIF-1α at lysine residue 709 by SIRT2 enhances recognition of hydroxylating residues by PHDs [17].

Reactive oxygen species (ROS) are also capable of inducing HIF-1α under normoxic conditions. Furthermore, exogenous hydrogen peroxide (H_2_O_2_) and glucose oxidase (GOx), which generates H_2_O_2_ can increase HIF-1α and HIF-2α stabilization [18]. Interestingly, the use of 2,3-dimethoxy-1,4-naphthoquinone (DMNQ), a redox cycler, has also been described to increase the levels of HIF-1α under normoxic conditions [18]. The mechanisms eliciting the increased levels of HIF-1α by ROS have been reported to be mediated by superoxide (SO) generated by xanthine/xanthine oxidase, which partially inhibited pVHL binding to HIF-1α [18]. Additionally, under normoxia, siRNA targeting of Mn-SOD has been shown to increase HIF-1α levels by inhibiting pVHL-HIF binding, which in turn inhibits the activity of PHDs [18]. There is also evidence that PHDs can be directly inhibited by the presence of SO [18].

On the other hand, nitric oxide (NO) has been reported to have a stimulatory effect over the accumulation of HIF-1α. Different studies have described that NO donors, macrophage-derived NO, NO synthase transfection, and increased endogenous NO via iNOS all induce HIF-1α accumulation and the transcription of HIF-1 target genes [18,19]. However, this effect has been shown to be concentration-dependent, as some concentrations of NO actually have an inhibitory effect over HIF-1α stabilization [18]. It has been reported that PI3K and Akt are essential signaling components in NO-induced HIF-1α stabilization [18]. Additionally, S-nitrosation may contribute to HIF-1α stabilization in a concentration-dependent manner [18]. Interestingly, S-Nitrosoglutathione (GSNO), which donates nitrosonium, induces S-nitrosation of HIF-1α and inhibits the interaction of pVHL with HIF-1 α in the presence of PHD1, PHD2, and PHD3 under normoxic conditions, and induces HIF-1α accumulation [18].

Importantly, different studies have reported a direct relationship between inflammatory diseases and hypoxia over the last decades. For instance, HIF-1α has been reported to play a relevant role in the function of immune cells, which may affect the host’s response to pathogens [20]. Additionally, this transcription factor has been reported to be involved in the outcome of many infections, such as those mediated by bacteria, parasites, and fungi [21,22,23]. Moreover, hypoxia may have a considerable effect over numerous viral infections, consistent with several viruses inducing a hypoxic response upon infection to promote their replication or stabilize HIF-1α with concomitant enhanced viral gene expression [24,25].

Below, we revise and discuss cellular responses to hypoxia and how viruses can modulate hypoxia-related genes in their favor.

## 2. Cellular Responses to Hypoxia

Numerous studies have detailed how hypoxic conditions induce a wide range of physiological and transcriptional changes in cells. Consistently, HIFs have been shown to target a wide array of genes with different functions and induce distinct cellular responses to hypoxic conditions, which are detailed below.

### 2.1. Regulation of Glucose Metabolism

**Figure 1:**
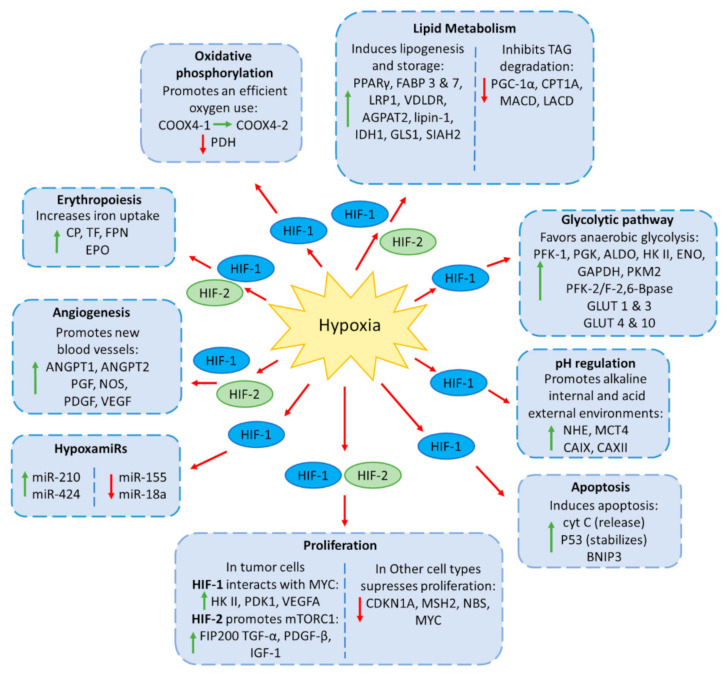
Cellular response to hypoxia. Under conditions of low oxygen levels, cells respond to hypoxia through hypoxia-inducible factors 1 and 2 (HIF-1 and 2), which modulate several key cellular processes. HIF-1 upregulates oxidative phosphorylation by interacting with COOX4 to promote efficient oxygen use, and pyruvate dehydrogenase (PDH) is inhibited. Lipid metabolism is also modulated by HIFs via upregulation of genes involved in lipogenesis and lipid storage and inhibition of genes involved in triacylglycerol degradation. Additionally, under hypoxic conditions, cells may favor anaerobic glycolysis through the upregulation of several genes involved in the glycolytic pathway. HIF-1 has also been related to the regulation of intracellular pH to promote an alkaline internal environment by inducing proton efflux transporters NHE and MCT4, and regulates carbonic anhydrases IX and XII (CAIX and CAXII). Apoptosis is also affected by hypoxia by increasing cytochrome c (cyt c) release, stabilization of p53, and inducing the BCL2 interacting protein 3 (BNIP3). Additionally, different effects of hypoxia over cell proliferation are induced by HIF-1α interaction with MYC leading to the expression of hexokinase 2 (HK II), pyruvate dehydrogenase kinase 1 (PDK1), and vascular endothelial growth factor A (VEGFA), and HIF-2 induces the rapamycin complex 1 (mTORC1) by inducing different growth factors. In contrast, cellular proliferation is suppressed when HIF-1α displaces MYC from target genes, such as CDKN1A, MSH2, and NBS1, and by reducing MYC promoter occupancy. Hypoxia has also been described to cause the specific expression of micro RNAs (HypoxiamiRs) involved in the tight regulation of HIF-1 function. Furthermore, angiogenesis is also affected by hypoxia to augment oxygen levels. HIF-1 and HIF-2 upregulate the expression of angiopoietin 1 and 2 (ANGPT1 and ANGPT2), platelet-derived growth factor B (PDGFB), nitric oxide synthase (NOS), placental growth factor (PGF), and vascular endothelial growth factor (VEGF). Lastly, erythropoiesis metabolism is influenced by HIF-1 and HIF-2, which cause an increase in the uptake of iron through the upregulation of transferrin (TF), ferroportin (FPN), and ceruloplasmin (CP), altogether promoting EPO production to boost erythrocyte production. Green arrows indicate induction or upregulation, while red arrows indicate inhibition or downregulation.

**Figure 2:**
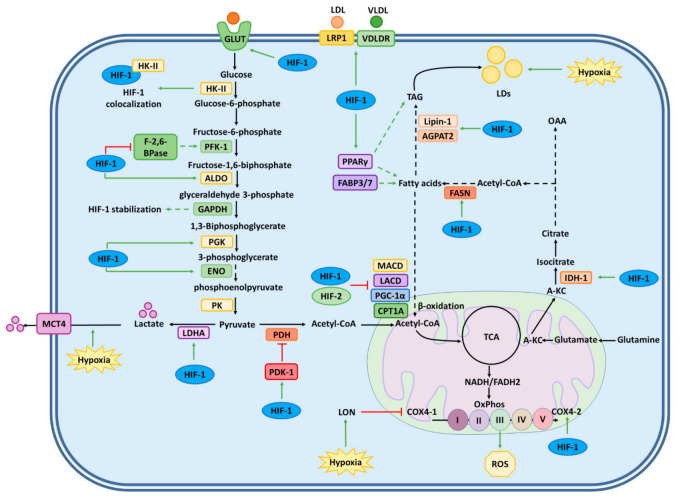
Metabolism regulation under hypoxia conditions. Cellular metabolism is affected by hypoxic conditions. Under low oxygen conditions, HIF-1 increases the transcription of enzymes involved in glucose catabolism. Similarly, HIF-1 enhances the anaerobic lactate pathway by increasing the transcription of lactate dehydrogenase A (LDHA). Furthermore, HIF-1 promotes the transcription of glucose transporters to offset low ATP availability. Conversely, pyruvate dehydrogenase kinase 1 (PDK-1) is induced, causing phosphorylation and inactivation of pyruvate dehydrogenase (PDH) to regulate the levels of acetyl-CoA. Additionally, HIF-1 upregulates isocitrate dehydrogenase-1 (IDH-1) and aconitase expression, causing a reduction in acetyl-CoA availability and reducing the activity of the tricarboxylic acid cycle (TCA) to decrease ROS generation by the mitochondria. Furthermore, hypoxia promotes the transcription of LON genes, inhibiting the expression of the Cytochrome c oxidase subunit 4 isoform 1 (COOX4-1). Altogether, HIF-1 upregulates COOX4-2, allowing a cellular environment for optimal use of oxygen during oxidative phosphorylation. Additionally, hypoxic conditions have been reported to affect lipid metabolism. Lipogenesis and lipids are upregulated by HIF-1 through the induction of several target genes, thus promoting fatty acid and triacylglycerol (TGA) synthesis. Additionally, endocytosis of lipoproteins is regulated during hypoxia through the overexpression of the low-density lipoprotein (LDL) receptor-related protein 1 (LRP1) and the very low-density lipoprotein (VLDL) receptor (VLDLR). Moreover, IDH1 is regulated by HIF-1 indirectly, which leads to maintaining fatty acid synthesis through reductive carboxylation. Lipid storage is upregulated by the induction of acylglycerol-3-phosphate acyltransferase 2 (AGPAT2) and lipin-1. Moreover, HIF-1 and HIF-2 inhibit enzymes that participate in fatty acid degradation via a downregulation in the expression of several target genes involved in this process. Green arrows indicate induction or upregulation, and red arrows denote inhibition or downregulation.

Cell metabolism in mammalian cells is highly dependent on oxygen, and thus, changes in the availability of this gas generate important changes in catabolic and anabolic processes. Under hypoxic conditions, cells will need to switch to anaerobic glycolysis to adapt to this scenario, consequently increasing glucose conversion to lactate [1]. In this regard, HIF-1, but not HIF-2, plays an important role in promoting the transcription of key enzymes involved in glucose metabolism. For instance, the transcription of glucose transporters (GLUT) GLUT1, GLUT3 and, to a lesser extent, GLUT4 and GLUT10, have been reported to be promoted by HIF-1 to offset low ATP availability [26,27]. An increase in the expression of GLUT transporters is positively correlated with the increased expression of other glycolytic enzymes, which suggests an interplay between HIF-1, GLUTs, and glucose-metabolism-related enzymes associated with host metabolic reprogramming in diseases such as cancer and those mediated by oncogenic viruses [28,29,30]. Glycolytic pathway enzymes, such as phosphofructokinase-1 (PFK-1), 6-phosphofructo-2-kinase/fructose-2,6-biphosphatase (PFK-2/F-2,6-BPase), phosphoglycerate kinase 1 (PGK-1), and lactate dehydrogenase A (LDHA), among others, are all upregulated by hypoxia through HIF-1 [31,32,33,34]. Furthermore, mRNA expression of hexokinase 2 (HK II), which catalyzes the phosphorylation of glucose to glucose-6-P, corelates with HIF-1α protein in hepatocellular carcinoma cells and metastatic liver cancer, and HK II and HIF-1α protein expression co-localized in these cancer cells [35,36]. Furthermore, during viral infections, such as those produced by the hepatitis C virus (HCV), HK II is overexpressed and in cells infected with the human respiratory syncytial virus (hRSV) HIF-1α suppression induced a decrease in HK II protein levels [37,38]. Moreover, PFK-1, which phosphorylates fructose-6-P to fructose-1,6 bisphosphate (FBP), is also activated under hypoxic conditions by HIF-1-induced PFK-2/F-2,6-BPase, and the downregulation of the latter in cancer cells has been reported to inhibit tumor growth [39,40]. Other genes encoding other glycolytic enzymes, such as aldolase (ALDO) and enolase (ENO), are also directly upregulated by HIF through HREs in their promoter regions [41]. Under hypoxic conditions, PGK1 and pyruvate kinase mammalian (PKM) catalyze the transfer of phosphoryl group to ADP, leading to ATP production [42]. Interestingly, PKM2, an isoform of PKM, has been described to act as a coactivator of HIF, and the expression of this glycolytic enzyme is induced by mTOR-mediated HIF-1 stabilization [43,44]. Finally, another target of HIF-1 in the glycolysis pathway is glyceraldehyde-3 phosphate dehydrogenase (GAPDH), which catalyzes the oxidation of glyceraldehyde-3 phosphate and reduction of NAD^+^ to NADH, and has been reported to be associated with enhanced aggressiveness of some tumors [45,46] (Figure 1 and Figure 2).

Additionally, hypoxia affects oxidative phosphorylation, where HIF-1 has been reported to modulate the cytochrome c oxidase subunit 4 isoform (COOX4), which allows a more efficient use of the available oxygen levels in the cell. A switch from COOX4-1 to COOX4-2 is elicited by the upregulation of the transcription of both the *COOX4-2* and *LON* (a mitochondrial protease required for COX4-1 degradation) genes, which ultimately leads to increased COOX4-2 protein synthesis and increased COOX4-1 proteolysis, respectively [47]. Moreover, pyruvate dehydrogenase (PDH) is inhibited through its phosphorylation by the pyruvate dehydrogenase kinase 1 (PDK1) under conditions of prolongated hypoxia by shunting pyruvate away from the mitochondria, thereby reducing the activity of the tricarboxylic acid cycle (TCA), as an adaptive response to prevent the generation of toxic levels of ROS [48]. HIF-1 will also redirect the use of glutamine towards citrate by upregulating isocitrate dehydrogenase-1 (IDH1) to maintain fatty acid synthesis through reductive carboxylation [49] (Figure 1 and Figure 2).

Importantly, hypoxia can induce acidosis due to an increase in lactic acid production by the cell [50,51]. Noteworthy, proteins associated with pH regulation are also targets of HIF. For instance, HIF-1 induces the expression of Na^+^/H exchanger 1 (NHE1), which mediates proton efflux from the cell [52]. Moreover, carbonic anhydrases IX and XII (CAIX and CAXII), which are also targets of HIF-1 [53], play essential roles in acidifying the extracellular environment, which is necessary to maintain an intracellular alkaline pH [50]. Additionally, the monocarboxylate transporter 4 (MCT4), which catalyzes proton-coupled transport of lactate, is induced by hypoxia (Figure 1 and Figure 2) [51].

### 2.2. Regulation of Lipid Metabolism

At present, there is accumulating evidence that indicates that lipid metabolism is modulated by HIF, and that this supports some cellular processes during hypoxia in healthy cells, as well as in tumors [54]. Importantly, the transcription factor PPARγ has been reported to be activated by HIF-1 and promote fatty acid (FA) and triacylglycerol (TAG) synthesis [55]. Moreover, the fatty acid-binding proteins (FABP) 3 and 7 are also upregulated by HIF and support lipogenesis in tumor cells [56]. Furthermore, endocytosis of lipoproteins is also regulated by HIF-1α under hypoxic conditions, through the overexpression of low-density lipoprotein receptor-related protein 1 (LRP1) and very low-density lipoprotein receptor (VLDLR) [57,58].

HIF-1 has also been reported to redirect glutamine towards citrate by an indirect upregulation of the IDH1 to maintain FA synthesis through reductive carboxylation (Figure 1 and Figure 2) [50]. In addition, α-ketoglutarate, which acts as a substrate for citrate production and FA synthesis, is also upregulated by HIF-1 through the induction of the expression of glutaminase 1 (GLS1) and E3 ubiquitin ligase SIAH2 [54,59]. Moreover, fatty acid synthase (FAS), which is the primary enzyme involved in lipogenesis, is also upregulated through Akt and HIF-1 activation under hypoxic conditions, leading to an Akt-mediated activation of the sterol regulatory element-binding transcription factor 1 (SREBP-1) [60].

On the other hand, TAGs have been reported to be stored in lipid droplets (LDs) during hypoxia through HIF-1 by inducing the expression of acylglycerol-3-phosphate acyltransferase 2 (AGPAT2) and lipin-1, which participate in these structures, as a mechanism to avoid lipotoxicity [61,62]. AGPAT2 mediates the conversion of lysophosphatidic acid (LPA) to phosphatidic acid (PA), which in turn is converted into diacylglycerol (DAG) by lipin-1; these two products are then used as precursors for TAGs [61,62]. Moreover, the accumulation of lipids within the cell during hypoxia is accompanied by the inhibition of enzymes that participate in FA degradation. In this regard, in cancer cells HIF-1 and HIF-2 have been reported to downregulate the expression of proliferator-activated receptor-γ coactivator-1α (PGC-1α), carnitine palmitoyltransferase 1A (CPT1A), and medium- and long-chain acyl-COA dehydrogenases (MACD and LACD), which are involved in β-oxidation inhibition [63,64].

### 2.3. Regulation of Erythropoiesis and Angiogenesis

Erythropoiesis and angiogenesis are also strongly influenced by hypoxia [65]. Interestingly, these processes are regulated both by HIF-1 and HIF-2 [65]. To meet iron needs for synthesizing hemoglobin, erythropoiesis induces changes in iron metabolism to increase its availability [66]. Importantly, HIFs upregulate the expression of genes involved in iron homeostasis, such as ceruloplasmin (encoded by the CP gene), which oxidizes Fe^2+^ to Fe^3+^, and transferrin (TF), which transports serum iron in its ferric form (Fe^3+^) [67,68,69]. On the other hand, both HIFs and hypoxic conditions can downregulate hepcidin (encoded by the HAMP gene), which results in increased cell surface expression of ferroportin (FPN) an iron exporter [70,71]. Additionally, erythropoietin (EPO) is also upregulated by HIFs [72], with this hormone playing an important role in producing red blood cells in hematopoietic organs (Figure 1) [65,70].

Angiogenesis is a process that is required during wound healing and inflammation for the generation of newly formed blood vessels. HIFs are known to target several genes involved in these processes [73,74,75]. For instance, HIF-1 induces the transcription of angiopoietin 1 and 2 (ANGPT1 and ANGPT2), placental growth factor (PGF), nitric oxide synthase (NOS), and platelet-derived growth factor B (PDGFB), among others [75,76,77]. Each of these proteins plays a specific role in forming new blood vessels, and thereby, the regulation of these factors is necessary for maintaining an optimal vasculature [1]. Interestingly, some of these factors, such as ANGPT-1, do not have known HRE sites, which has led to the belief that HIF indirectly induces these factors under hypoxic conditions [77]. Additionally, vascular endothelial growth factor (VEGF), which directs the migration of mature endothelial cells towards hypoxic areas, is also induced by hypoxia [78]. HIF-1 activates VEGF by directly binding to an HRE present in the *VEGF* gene [78]. Furthermore, it has been described that multiple VEGF receptors are also regulated by hypoxia [79]. Additionally, a study reported that HIF-1 induces an extracellular matrix invasion and tube formation via inducing autonomous endothelial cell activation (Figure 1) [80].

### 2.4. Regulation of Apoptosis and Cell Proliferation

Apoptosis and cell proliferation are two cellular processes that are regulated, among others, by hypoxia. Indeed, hypoxia induces the hyperpermeability of the inner mitochondrial membrane, which causes cytochrome c release, and consequently the inhibition of the electron transport chain, which decreases membrane potential and reduces mitochondrial-derived ATP production [81,82]. This, in turn, activates BCL2 associated X protein (Bax) causing cyt c release to the cytosol [82]. In a chemically-induced apoptosis model, it has been shown that cells that lack VHL are sensitive to apoptosis but that the reintroduction of this protein renders the cell resistant to apoptosis [81,83]. Furthermore, it has been reported that under hypoxic conditions, HIF-1α can interact with and stabilize p53, which induces programmed cell death by regulating proteins such as Bax [81,84]. This interaction was particularly shown to be mediated by the mouse double minute 2 homolog (Mdm2), a p53 ubiquitin ligase that negatively regulates p53 [81,84]. Interestingly, HIF-1α has been described to upregulate p53 levels by inhibiting Mdm2-mediated degradation, while HIF-2α has been reported to have a contrary effect over p53, by inhibiting this protein in an Mdm2-independent manner [81,84,85]. Moreover, a relation between HIF-1 and the proapoptotic BCL2 interacting protein 3 (BNIP3) has also been established [81]. This latter protein, which can bind and inhibit the anti-apoptotic proteins Bcl-2 and Bcl-xL, was reported to be upregulated under hypoxic conditions in different cell lines [86,87]. Additional data supporting the notion that upregulation of apoptosis under hypoxic conditions may occur in a HIF-1α-dependent manner is supported by the finding that cells lacking HIF-1 are unable to produce a large amount of BNIP3, which correlates with reduced cell death rates [87]. Interestingly, the promoter of BNIP3 contains a HRE [81,88].

Different studies support the notion that cellular proliferation is suppressed by hypoxia in several cell types and, more specifically, that HIF-1α stabilization under hypoxic conditions produces cell cycle arrest through the inhibition of the pro-oncoprotein MYC [89]. Importantly, this protein controls G1/S cell cycle transition and inhibits the expression of p21 and p27, and MYC promotes cellular proliferation by upregulating the expression of glycolytic enzymes and protein synthesis, thus promoting cell growth [89,90]. Interestingly, it has been reported that HIF1α binds to the SP1 transcription factor and displaces MYC from multiple target genes, such as CDKN1A, MSH2, and NBS1 [89,91,92]. Furthermore, it was found that HIF-1α can disrupt the association between MYC and the myc-associated factor X (MAX), and Myc-interacting zinc finger protein 1 (MIZ1), which causes a reduction in MYC promoter occupancy in several target genes [89,93]. Furthermore, under chronic hypoxia, it was reported that HIF-1α promoted MYC degradation [94,95]. Intriguingly, transformed cells expressing HIF-2α exhibit enhanced MYC activity, a rapid entry into the S phase of the cell proliferation cycle, and increased MYC promoter occupancy [89,93]. Additionally, HIF-2α may stimulate the target of rapamycin complex 1 (mTORC1), leading to the induction of cellular proliferation under hypoxic conditions [96]. In this study, it was found that the upregulation of mTORC1 by HIF-2α occurs through the induction of the focal adhesion kinase (FAK) family interacting protein of 200 kD (FIP200) gene, which is a HIF-2α target gene [96]. In turn, FIP200 interacts with tuberous sclerosis complex 1 (TSC1), which induces the disruption of the TSC1-TSC2 complex, leading to mTORC1 activation [96]. Additionally, it was found that HIF-2α could also upregulate mTORC1 activity by inducing the expression of growth factors, such as the transforming growth factor-alpha (TGF-α), platelet-derived growth factor subunit B (PDGFB), and IGF-1, which lead to Akt and mTORC1 activation [97].

Interestingly, the expression of MYC in tumors and its interplay with HIF-1α seem to play an important role in cellular proliferation in transformed cells. High levels of MYC have been reported to sequester and bind to MAX, causing relief in the inhibition by HIF-1α [98]. Additionally, some findings support the notion that HIF-1α can cooperate with MYC to induce the expression of certain genes, including hexokinase 2 (HK II), pyruvate dehydrogenase kinase 1 (PDK1), and vascular endothelial growth factor A (VEGFA) [98]. For a more comprehensive view on the effects of HIFs and hypoxia over tumor cell proliferation, we suggest reading the reviews written by Keith et al. [89] and Gordan et al. [99].

### 2.5. miRNAs Regulated by Hypoxia

Several miRNAs have been described to be involved in the cellular response to hypoxia [100,101]. miRNAs are defined as small non-coding RNA molecules of approximately 22 nucleotides [101]. These molecules usually bind to the 3′UTR of mRNAs and modulate their stability and translation, generally reducing translated protein levels [102]. Therefore, they may control HIF expression under hypoxic conditions.

Several miRNAs regulated by hypoxia (hypoxamiRs) have been described, and many of these are tissue-specific, except for the “master hypoxamiR” miR-210, which is robustly and ubiquitously induced by cells regardless of the tissue [103].

Interestingly, such miRNAs may contribute to inducing positive or negative feedbacks to the stabilization of HIFs. For instance, miR-210 induces a positive feedback loop with HIF-1α, with this factor driving miR-210 expression, which inhibits the activity of PHDs, which are the most commonly described HIF-1α inhibitors, thus increasing HIF-1α stabilization [104,105]. Additionally, miR-424 has also been reported to elicit a positive feedback loop with HIF-1α, as endothelial cells under hypoxic conditions express this hypoxamiR, which was shown to inhibit cullin 2, a protein necessary for the assembly of the ubiquitin ligase system, thereby stabilizing HIF-1α [106] (Figure 1).

In contrast, miR-155, which is also induced by HIF-1α, binds to the mRNA of *HIF-1A* and consequently decreases HIF-1 protein levels [107]. Furthermore, miR-18a has been reported to be upregulated by hypoxia in human endothelial cells and also targets *HIF-1A* mRNA, thus reducing HIF-1α protein levels [108]. Interestingly, members of the miR-17/92 cluster may also target *HIF-1A* in a direct and dose-dependent manner. However, HIF can suppress the expression of this miRNA under hypoxic conditions (Figure 1) [109]. Altogether, the role of miRNAs and their relationship with the cellular responses to hypoxia is intricate and has been extensively reviewed by Bertero and Serocki [100,101].

Taken together, multiple key cellular pathways are directly affected and modulated by hypoxia, which in turn causes a wide range of cellular responses oriented at adapting to these new adverse conditions. In this context, HIFs play a key role as master regulators of numerous physiological changes under low oxygen levels. Interestingly, the study of these transcription factors and their relationship with multiple cellular responses opens the opportunity for targeting these transcription factors as possible candidates for treating different diseases. For instance, VEFG and ANGPT-1 play an important role in ischemic diseases as they are able to stimulate the remodeling of collateral blood vessels, leading to increased blood flow for different tissues [110], and their regulation via HIF is an interesting approach for developing new therapeutic strategies.

## 3. Effect of Hypoxia over Viral Infections

During the last years, numerous studies have reported a relationship between viruses and hypoxia [25]. However, this response varies widely among viruses, as in some cases hypoxia may promote viral replication, while others downregulate this process [24,25].

For instance, hypoxic states have been reported to aid the Epstein–Barr virus (EBV, HHV-4) and the Kaposi’s Sarcoma-associated herpesvirus (KSHV, HHV-8) switching from a latent to a lytic mode. Indeed, low oxygen levels have been shown to increase the expression of Zta, a protein that mediates this switch between latent and lytic infection in EBV in a B-lymphoblastoid cell line [111]. This, in turn, increased the number of copies of the viral DNA in the infected cells (Table 1) [111]. Additionally, HIF-1α has been described to induce EBV lytic gene expression by binding to the promoter of the latent-lytic switch *BZLF1* gene Zp [112].

Moreover, HIF-1α has been reported to induce the expression of KSHV lytic genes. Under hypoxic conditions, HIF-1α binds to the latency-associated nuclear antigen (LANA) of KSHV. In turn, the HIF-1α:LANA complex binds to an HRE present in the promoter region of the viral *Rta* gene, eliciting increased expression of its product and playing an important role as a lytic replication and transcriptional activator [113]. Likewise, hypoxia induces the transcription of ORF34 in KHSV, a lytic gene, namely through HIF-1α or HIF-2α, which bind to an HRE located in the promoter region of the corresponding gene (Table 1) [114].

Furthermore, it has been shown that under hypoxic conditions, HIF-1α can elicit a metabolic reprogramming in KSHV-infected B cells by inducing vGPCR (G protein-coupled receptor), which is considered as a lytic gene needed for KSHV viral reactivation [127]. This protein has also been described as a potent candidate in the upregulation of several cell proliferation pathways, such as MAP kinase and angiogenesis [128]. Additionally, this protein plays an important role in tumorigenesis due to its capacity to activate MEK/ERK pathways [128]. Furthermore, the expression of vGPCR is associated with global transcriptional regulation, the generation of ROS, and enhanced glucose uptake [127]. Similarly, hypoxia induces lytic replication of KSHV through the viral inducer 12-O-tetradecanoylphorbol-13-acetate (TPA) [115], which elicits an increase in interleukin-6 (IL-6) levels and stimulates spindle cell growth while likely activating angiogenic factors (Table 1) [115].

Interestingly, an oncolytic mutant herpes simplex virus type 1 (HSV-1 G207) with a deletion in the γ34.5 gene has been reported to display increased replication under hypoxic conditions [116]. This phenomenon would be partly due to an increase in the expression of GADD34, a mammalian factor that can complement the mutation, particularly under hypoxic conditions (Table 1) [116].

The John Cunningham virus (JCV) has also been reported to be affected by hypoxic conditions. HIF-1α has been shown to bind to the JCV non-coding regulatory region (control region) in the viral genome and activate early and late viral gene promoters, which may help JCV replicate (Table 1) [117].

Hypoxia has also been described to upregulate infection of some RNA viruses. For instance, genome replication of the dengue virus (DENV), but not virus entry or RNA translation, was enhanced under hypoxic conditions and was mediated through HIF-1α/2α, the serine/threonine kinase AKT, and ROS in liver cells, monocytes and epithelial cells (Table 1) [120].

Additionally, it has been reported that the activity of the promoter of the human immunodeficiency virus (HIV) is upregulated by a heterodimer formed by the viral protein Vpr and HIF-1α, which bind to the GC-rich binding domains in the long terminal repeat (LTRs) [121]. Furthermore, under hypoxic conditions, an interleukin-7 (IL-7)-induced increase in the expression of the GLUT1 has been observed, which leads to an increase in glucose uptake favoring HIV-1 infection [122] (Table 1). On the other hand, replication of the vesicular stomatitis virus (VSV) is higher under low oxygen levels than normoxic conditions, when using a tumor environment and when HeLa cells are cultured under hypoxic conditions. This showed that early after infection, viral mRNAs are reduced, but later on, VSV can surpass the inhibition of viral protein synthesis by overcoming the hypoxia-associated increase in the phosphorylation of the eukaryotic initiation factor 2α (eIF-2α) (Table 1) [124].

In contrast, some viruses have been described to be downregulated under hypoxic conditions. For instance, low oxygen levels have been reported to induce a reduction in the expression of the adenovirus protein E1A through a post-transcriptional mechanism [118]. Importantly, this protein is necessary for driving the cell into the S phase of the replication cycle of cells, thus suggesting a hypoxia-induced G1 arrest of cells that leads to reduced viral replication (Table 1) [118]. Interestingly, another study showed that E1A mRNA levels are not affected under hypoxic conditions. Thus, a reduction in E1A protein levels under hypoxic conditions may occur without reducing E1a mRNA levels [129].

Additionally, it has been reported that the angiotensin-converting enzyme 2 (ACE2), a cellular receptor key for the infection process mediated by severe acute respiratory syndrome coronavirus 2 (SARS-CoV-2), and which mediates viral entry into the target cells, is decreased upon HIF-1α accumulation, which could translate into lesser infection and viral replication [126,130]. This phenomenon may occur given that hypoxic conditions increase the levels of angiotensin II, which in turn inhibits the synthesis of ACE2 [125]. Consequently, populations that live in high altitudes and are chronically exposed to hypoxic conditions seem to exhibit minor pathology after SARS-CoV-2 infection [131]. Another study also supports the role of HIF-1α over the replication cycle of SARS-CoV-2, with mRNAs and proteins of viral receptors, ACE2 and TMPRSS2, being downregulated in cells cultured under hypoxic conditions [125]. Moreover, this study evaluated the effect of HIF-1α after viral entry by inducing hypoxia 8 h post-infection, which showed a 90% reduction of viral RNA levels, and decreased the release of viral particles compared to during normoxic conditions (Table 1) [120]. In contrast, other studies have reported the expression of the *ACE2* gene by hypoxia [126,132,133], which may be explained by regulations at the transcriptional or translation level and may be cell type specific and may also depend on the existence of metabolic phenotypes that modulate HIF signaling [134].

Furthermore, under hypoxic conditions, the deoxyribonuclease 1 gene (*Dnase 1*) is overexpressed and encapsidated in hepatitis B virus (HBV) particles, which induced viral DNA degradation and thus promoted the formation of genome-free HBV virions (Table 1) [119].

Interestingly, downregulation of HIV-1 replication under low oxygen conditions has also been reported. However, this reduction was not due to HIF-1α expression, as it was transitory at 1% O_2_ concentration and absent at 3% O_2_. Noteworthy, HIV transcription is induced by the viral protein Tat and under conditions of 3% O_2_, and this process was found to be reduced compared to a situation with 21% O_2_ [123]. This reduction observed at 3% O_2_ is suggested to be mediated by a decreased activity of the holoenzyme complex CDK9/cyclinT1, and the transcription factor Sp1 (Table 1) [123].

Noteworthy, heme oxygenase-1 (HO-1), an inducible enzyme expressed in response to physical and chemical stress, has been shown to be induced under hypoxic conditions. Human dermal fibroblasts that undergo hypoxia display a strong stabilization of HO-1 mRNA [135]. Additionally, different studies describe that HO-1 is stimulated by hypoxia in several cell types, such as astrocytes, cardiomyocytes, Chinese hamster ovary cells (CHO), and vascular smooth muscle cells [136,137,138,139]. Interestingly, pharmacological induction of HO-1 by cobalt protoporphyrin (CoPP) has been reported to impair the propagation of herpes simplex virus type 2 (HSV-2) in vitro [140]. Similarly, HO-1 induction by CoPP has been reported to inhibit the replication of the human respiratory syncytial virus (hRSV) and lung inflammation in vivo [141]. Other viruses are also affected by HO-1 expression [141,142]. Thus, hypoxia may elicit antiviral effects through other host factors, such as HO-1.

Taken together, hypoxia not only has a significant impact on the regulation of key cellular responses but also modulates infection mediated by several human viral pathogens. Interestingly, pathogens may benefit from such low oxygen levels or the cell responses elicited under these conditions. Indeed, hypoxia affects the replication cycle of several viruses and may promote their replication or activation from latent states eliciting lytic gene expression. Importantly, hypoxic conditions or the induction of cellular responses to hypoxia can also negatively impact viral processes, such as some related to SARS-CoV-2. Interestingly, Vadadustat, an α-ketoglutarate analog that acts as a PDH inhibitor and thus is capable of stabilizing HIF-1α, is currently in clinical trials to treat acute respiratory distress syndrome (ARDS) in COVID-19 patients (https://clinicaltrials.gov/ct2/show/NCT04478071 (accessed on 4 July 2021)). Thus, hypoxia and the cellular response elicited to this condition arises as an attractive approach to explore the development of new antiviral treatments. We foresee that more clinical trials evaluating the use of drugs capable of regulating the expression of HIF-1α are likely to occur in the near future due to the accumulating evidence available regarding the interplay between hypoxia and human viral infections.

## 4. Virus Induction of Hypoxia Responses

**Figure 3:**
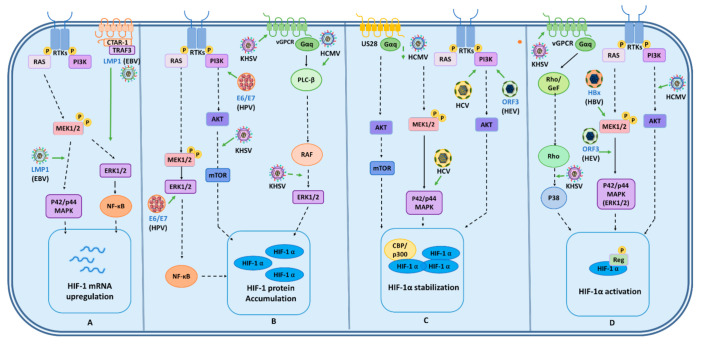
Viral modulation of kinase-dependent hypoxia responses. Different kinase signaling cascades lead to the activation of HIF-1⍺. (**A**) The EBV latent membrane protein 1 (LMP-1), through CTAR-1, activates TRAF3, which activates the p44/p42 MAPK pathway and mediates the recruitment of ERK1/2/NF-κB to the promoter of the HIF-1A gene resulting in the induction of HIF-1⍺ transcription. (**B**) KHSV, through vGPCR is capable of inducing ERK1/2 and AKT signaling pathways, increasing HIF-1⍺. HPV type 16 oncoproteins E6 and E7 induce HIF-1⍺ protein accumulation by activating the ERK1/2 and PI3K/AKT pathways. (**C**) Hepatitis viruses have also been reported to have the capacity to activate kinases to upregulate HIF-1⍺. For instance, to promote HIF-1⍺ stabilization, HCV and the ORF3 protein of HEV can activate the PI3K/AKT pathway, while the ORF3 protein of HEV also induces ERK1/2-dependent phosphorylation of CBP/p300 to transactivate HIF-1⍺. Moreover, HCMV induces the upregulation of HIF-1⍺ stabilization through the viral chemokine receptor US28, by modulating a Gαq calcium/calmodulin-dependent protein kinase II (CaMKII). (**D**) To promote HIF-1⍺, HBV virus protein X (HBx) induces the nuclear translocation and activation of HIF-1⍺, by activating the MFK1/2 ERK pathway. On the other hand, HCMV may activate HIF-1α through the PI3K/AKT/mTOR pathway. Similarly, KHSV has been shown to upregulate vGPCR, and induce the paracrine activation of the alpha subunit of a Gq protein receptor, which promotes p38-dependent phosphorylation of the regulatory domain of HIF-1⍺ and its concomitant upregulation. EBV: Epstein–Barr virus; KHSV: Kaposi’s Sarcoma-associated herpesvirus; HPV: Human papillomavirus; HCV: Hepatitis C virus; HEV: Hepatitis E virus; HBV: Hepatitis B virus; HCMV: Human cytomegalovirus.

**Figure 4:**
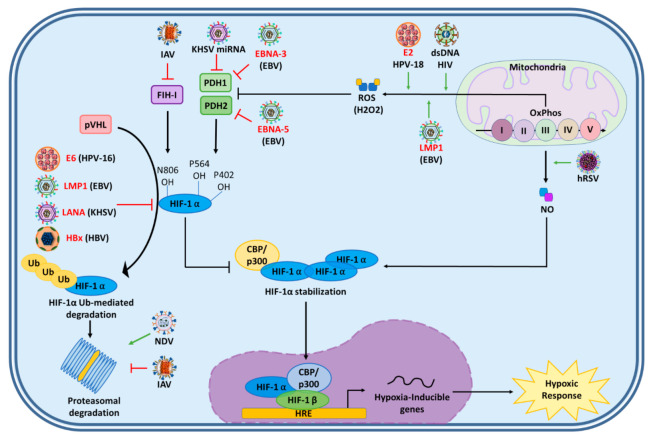
Viral modulation of HIF-1⍺ inhibitors. Several viruses have been reported to activate HIF-1⍺ and thus, promote the expression of hypoxia-inducible genes that are part of the hypoxic response. This is accomplished by the stabilization of HIF-1⍺ and avoidance of its proteasomal degradation. The E6 oncolytic protein from HPV-16, LMP-1 from EBV, LANA from KHSV, and HBx from HBV have all demonstrated the ability to inhibit HIF-1⍺ ubiquitination and target this protein to the proteasome. On the other hand, the influenza A virus has been reported to be able to directly inhibit the activity of FIH-1, while also inhibiting the proteasome. On the other hand, KHSV miRNAs inhibit PHD1 expression, while the EBV EBNA-3 and EBNA-5 proteins inhibit PHD1 and PHD2, respectively, promoting HIF-1⍺ stabilization. Lastly, hRSV, the HPV-18 E2 protein, the EBV LMP-1 protein, and dsDNA from HIV have been described to increase ROS production by the mitochondria (the former virus through NO, while the other through H2O2), which regulate HIF-1 stabilization by inhibiting PHDs function. Contrarily to the viruses mentioned above, NDV was found to promote proteasomal degradation of HIF-1⍺. EBV: Epstein–Barr virus; KHSV: Kaposi’s sarcoma-associated herpesvirus; HPV: Human papillomavirus; HBV: Hepatitis B virus; HIV: Human immunodeficiency virus: IAV: Influenza A virus; hRSV: Human respiratory syncytial virus; NDV: Newcastle disease virus; PHD: prolyl-hydroxylase domain proteins; ROS: reactive oxygen species; NO: Nitric oxide.

Several studies have reported an upregulation of HIF-1α during viral infections [25]. However, the mechanisms underlying viral modulation of this transcription factor are quite variable and depend on several factors, which are depicted in Figure 3 and Figure 4 and described below.

### 4.1. Activation of HIF-1α Mediated by Viral Kinases

Different studies have reported that viruses may activate HIF-1α via kinases. For instance, a high-risk type of human papillomavirus (HPV) oncoproteins, E6 and E7 of the HPV type 16 (HPV-16), have been described to induce cellular HIF-1α protein accumulation by activating the ERK1/2 and the PI3K/Akt signaling pathways [143] (Figure 3).

Additionally, the latent membrane protein 1 (LMP1) of EBV has been reported to increase HIF-1α expression through the p44/42 MAPK pathway [144]. Moreover, this viral protein induced the promoter activity of HIF1A, which was accomplished by LMP1 through the recruitment of the C-terminal activating region 1-(CTAR-1) of ERK1/2/NF-κB and consequently led to the induction of the transcription of the HIF-1α gene (Figure 3) [145].

Furthermore, KSHV induction of HIF-1α is also mediated by kinases. Paracrine activation of mTOR has been reported to promote the upregulation of HIF-1, which was accomplished by viral GPCR activation through multiple signaling pathways, such as those associated with ERK and AKT [146]. These pathways, in turn, stimulate tuberin 1 and 2 (TSC1 and 2), leading to a derepression of mTOR and, thereby, to the induction of HIF-1α [146]. Moreover, vGPCR was shown to be able to induce the phosphorylation of the HIF-1α regulatory/inhibitory domain by the signaling pathways of p38 and mitogen-activated protein kinase (MAPK) leading to an upregulation of this transcription factor (Figure 3) [146].

Additionally, the human cytomegalovirus (HCMV or HHV-5) has been described to increase the stabilization of HIF-1α through its chemokine receptor US28 which stimulates the Gq protein alpha subunit (Gαq)-dependent signaling cascades leading to calcium/calmodulin-dependent protein kinase II (CaMKII)-activation and subsequent stimulation of AKT/mTOR [147]. Interestingly, the activation of HIF-1α after HCMV infection has also been reported through the PI3K/AKT pathway (Figure 3) [148].

Hepatitis B, C, and E viruses (HBV, HCV, and HEV, respectively) also induce HIF-1α activation through kinases. For instance, HBV protein X (HBx) induces the nuclear translocation and transcriptional activation of HIF-1α by activating the MEK1/p42/p44 MAPK pathway, which induces the transcriptional activation and nuclear translocation of HIF-1α [149]. HCV activates PI3K/Akt and p44/42 MAPK pathways leading to HIF-1α stabilization [150]. Additionally, the ORF3 protein of HEV increases HIF-1α stabilization through PI3K-mediated activation of AKT/protein kinase B and increases the transactivation activity of HIF-1 via ERK activation and CBP/p300 phosphorylation (Figure 3) [151].

### 4.2. Activation of HIF-1α Mediated by Viral Impairment of HIF-1α Inhibitors

HIF-1α stabilization requires the inhibition of molecules such as pVHL ubiquitin E3 ligase complex, PHDs, or FIH-1. Because of the relevance of these interactions, several viruses attack this pathway as a strategy to induce HIF-1α upon infection. For instance, the HPV-16 oncoprotein E6 enhances HIF-1α activation by protecting it from proteasomal degradation [152]. To accomplish this, E6 attenuates pVHL binding to HIF-1α, and consequently promotes HIF-1α accumulation (Figure 4) [152].

Furthermore, the EBV viral protein LMP1 upregulates Siah1 E3 ubiquitin ligase, leading to proteasomal degradation of PHD1 and PHD3, thus inhibiting the formation of the pVHL/HIF-1α complex ultimately promoting HIF-1α stabilization [153]. It has also been shown that two EBV nuclear proteins, EBNA-3 and EBNA-5, bind to PHD2 and PHD1, respectively, reducing their enzymatic activity [154]. This, in turn, inhibits PHD hydroxylation of HIF-1α and its degradation (Figure 4) [154].

Additionally, KSHV’s LANA has been described to function as a component of the EC_5_S ubiquitin complex and target VHL (the pVHL coding gene) and p53 for degradation, and therefore inhibit pVHL- and p53-mediated HIF-1α degradation [155]. Interestingly, miRNAs encoded in the KSHV genome downregulate the expression of PHD1, which leads to the stabilization of HIF1α (Figure 4) [156].

Moreover, the influenza A virus (IAV) has been reported to stimulate the inhibition of the proteasome. Furthermore, decreases in the expression of FIH-1 inhibit the degradation of HIF-1α, leading to its stabilization (Figure 4) [157].

Finally, the HBV protein X has been shown to stimulate HIF-1α protein stabilization by inhibiting the pVHL-mediated proteasomal degradation pathway [158]. Furthermore, this viral protein causes the deacetylation of the oxygen-dependent degradation domain of HIF-1α [159]. This, in turn, induces the dissociation of PHDs and pVHL from HIF-1α, leading to the stabilization of this transcription factor [159]. This process is probably mediated by the metastasis-associated protein 1 (MTA1) and histone deacetylases (HDAC), with their expression being enhanced by HBx (Figure 4) [159].

### 4.3. Activation of HIF-1α by Reactive Oxygen Species

Some viruses have also been reported to activate HIF-1α through ROS, NO, H_2_O_2_.

For instance, the E2 viral protein of human papillomavirus type 18 (HPV-18) has been reported to localize to mitochondrial membranes and induce the production of mitochondrial ROS without causing cell death [160]. The presence of these ROS correlated with the stabilization of HIF-1α and the induction of HIF-1 target genes (Figure 4) [156].

Additionally, it has been shown that the induction of HIF-1α by the EBV LMP1 protein is mediated through H_2_O_2_ production [144]. This was shown in LMP1-transfected Ad-AH cells treated with catalase, an H_2_O_2_ scavenger that strongly suppresses LMP1-induced production of H_2_O_2_. HIF-1α induction, in this case, was completely blocked upon this treatment (Figure 4) [144].

Furthermore, it has been shown that the human respiratory syncytial virus (hRSV) can stabilize HIF-1α through NO. Human bronchial epithelial cells infected with this virus release NO, which was reported to stabilize HIF-1α. Consistently, the inhibition of NO blocked the expression of HIF-1α and HIF-1 target genes in these cells (Figure 4) [161].

Moreover, cytosolic double-stranded DNA, generated during HIV replication in CD4^+^ T cells, has been described to induce mitochondrial ROS-dependent HIF-1α stabilization [162]. Additionally, the viral protein Vpr was described to induce ROS by increasing H_2_O_2_, which in turn leads to HIF-1α stabilization upon HIV infection (Figure 4) [163].

In summary, hypoxic cellular responses and viral infections have a bidirectional relationship, with hypoxia upregulating several processes related to the replication cycle of viruses and with viruses being able to induce a hypoxic response in the infected cells. Interestingly, at present, multiple studies are describing how viruses target different cellular pathways to upregulate HIF-1α. Importantly, the stabilization of this transcription factor may positively affect the infection of viruses and thus opens the possibility of pharmacological inhibition of HIFs as new antiviral treatments.

## 5. Virus Inhibition of Hypoxic Responses

### Viruses Downregulating HIF-1α

Interestingly, there is also evidence that some viruses may downregulate the stabilization of HIF-1α. For instance, the Newcastle disease virus (NDV) has been described to downregulate HIF-1α through its targeting to the proteasomal pathway and inhibit HIF-1α protein accumulation in various cell lines upon infection, overall producing a decrease in the transcription of HIF-1-target genes (Figure 4) [164].

Additionally, infection of reovirus-permissive tumor cells and reovirus-resistant tumor cells with the mammalian orthoreovirus (MRV, reovirus) was described to downregulate HIF-1α protein levels. Interestingly, this effect was also induced by UV-inactivated virus, which indicates that this downregulation is independent of virus replication [165]. Moreover, transfection of reovirus genome into human tumor cell lines reduces HIF-1α protein levels, even in the presence of polyinosinic-polycytidylic acid (polyI:C), which is a synthetic double-stranded RNA analog that acts as a pathogen-associated molecular pattern (PAMP), thus indicating that viral RNA plays a key role in the downregulation of HIF-1α [165].

Taken together, some viruses are also able to downregulate the hypoxic cellular response and modulate the effect of HIF-1α, thus overall impacting viral infections and varying their impact in the host. This observation opens the possibility for new studies that may focus on assessing how the stabilization of this transcription factor by different host and viral molecules or by mimicking hypoxia may contribute to viral control and treatment.

## 6. Conclusions

Cellular oxygen sensing mechanisms are essential for helping cells regulate critical functions during hypoxic situations and modulating the outcome of different diseases. The studies revised and discussed in this review highlight the key role of HIF transcription factors in guaranteeing homeostasis related to multiple cellular processes through the modulation and the expression of sets of genes that encode components related to responding to hypoxia.

Notably, at present, there is accumulating evidence suggesting that oxygen-sensing components in the cell and their response to varying oxygen levels can significantly affect viral infections, either by directly impacting their replication cycles or by regulating the immune responses elicited by them. This relationship between viruses and the hypoxic cellular response suggests potential pharmacological modulation of key oxygen-sensing components, such as HIF-related pathways, as therapeutic targets against several viruses and the diseases they cause. However, special care must be taken in defining whether HIF stabilization or inhibition exerts beneficial or harmful effects in response to viral infection, as HIF may promote viral replication in some cases, whereas it can inhibit this process in other situations. Therefore, more studies are needed to understand how different viruses respond to hypoxia-related factors and how viruses may modulate oxygen-sensing pathways to their favor.

It is crucial that in vitro studies take into account oxygen microenvironments since not all organs receive the same amount of oxygen in the organism, and changes in oxygen levels may have different effects over viruses depending on viral cell tropism as hypoxia may promote the replication of viruses in some cells or inhibit their fitness in others. Therefore, refined studies and experimental settings could aid in translating the findings into effective antiviral therapies.

Currently, HIF1α inhibitors are being tested in clinical trials to treat cancers and anemia, among others. These could be assessed as potential antiviral drugs or as adjuvant therapies to traditional treatments that are not fully effective. It is important to note that before the COVID-19 pandemic, no ongoing clinical trials had evaluated the potential therapeutic effects of HIF-1 stabilizers in viral infections. However, in the search for new antivirals to combat SARS-CoV-2, this situation has accelerated multiple investigations in this regard in humans. Vadadustat, an α-ketoglutarate analog, is currently being evaluated in clinical trials to treat acute respiratory distress syndrome (ARDS) in COVID-19 patients (https://clinicaltrials.gov/ct2/show/NCT04478071) (first posted on 20 July 2020). Interestingly, because MRV is known to inhibit HIF-1α, infection with this virus has been evaluated as a potential treatment for cancer [166]. Several studies report that MRV inhibits the accumulation of HIF-1α in infected cells and regulates gene expression in tumors in vitro and in vivo [166,167,168]. Furthermore, a Phase I clinical trial to evaluate the safety of treatment with this virus has provided satisfactory results and allowed the initiation of Phase II and Phase III clinical trials against multiple tumor types [166,169]. However, data so far from clinical trials have shown that this MRV-based therapy does not inhibit the growth or regression of all types of tumors [166,169]. Thus, further preclinical and clinical trials are needed to establish the potential of MRV as an effective treatment for different types of cancer.

In the short term, this approach should provide valuable information on whether using this type of procedure works as a new or additional therapy for treating viral infections. Furthermore, this strategy could be beneficial for treating chronic viral diseases, increasing the availability of drugs to be used, which is urgently needed for many viruses, particularly for those displaying resistance to conventional antiviral therapies.

## Figures and Tables

**Table 1:** Hypoxia effect over viral infections.

Virus	Cellular Process Modulated	Outcome	Model	References
**DNA Viruses**				
Epstein–Barr virus (EBV)	Hypoxia increases the expression of the EBV transcription factor Zta in a B-lymphoblast cell line.	Increases EBV replication.	In vitro	[111]
	HIF-1α interacts with the latent-lytic switch BZLF1 gene Zp in HEK 293T cells.	Increase EBV lytic gene expression.	In vitro	[112]
Kaposi’s Sarcoma-associated herpesvirus (KSHV)	HIF-1α binds to the latency-associated nuclear antigen (LANA) of KSHV. The HIF-1α-LANA complex induces *Rta* gene transcription in primary effusion lymphoma (PEL) cells.	Increases KSHV replication.	In vitro	[113]
	HIF-1α interacts with the promoter of the viral ORF34 gene in Hep3B cells and induces vGPCR expression in B cells.	Increase KSHV replication.	In vitro	[114]
	Hypoxia induces the expression of the viral inducer TPA, producing an increase in the levels of IL-6 expression in KSHV-infected primary effusion lymphoma B-cell lines.	Increase KSHV replication.	In vitro	[115]
Herpes simplex virus type 1 (HSV-1) mutant G207 (γ34.5 gene deletion)	Hypoxia increases GADD34 expression in U87 human glioma cells.	Increase of HSV-1 mutant G207 replication.	In vitro	[116]
John Cunningham virus (JCV)	HIF-1α interacts with the JCV control region in glial cells.	Increases JCV replication.	In vitro	[117]
Adenovirus	Hypoxia decreases the expression of adenovirus protein E1A.	Decreases adenovirus replication in HEK293 and numerous cancer cell lines.	In vitro	[118]
HBV	Hypoxia induces DNase 1 expression, which is encapsidated in HBV particles.	HBV genome copy number reduction in HepG2 cells.	In vitro	[119]
**RNA Viruses**				
DENV	HIF-1α and HIF-2α induce serine/threonine AKT signaling and ROS production in liver cells.	Increase DENV replication.	In vitro	[120]
HIV	HIF-1α interacts with VPR inducing HIV gene expression in human kidney cells.	Increases HIV production.	In vitro	[121]
	Hypoxia induces IL-17 expression that upregulates GLUT1 expression, favoring glucose uptake in T cells.	Increases HIV production.	In vitro	[122]
	Hypoxia decreases CDK9/cyclin T1 and Sp1 expression, producing a reduction in HIV Tat expression.	Reduction in HIV production.	In vitro	[123]
VSV	Hypoxia reduces VSV mRNA at early time points after infection of HeLa cells, but at later times after infection, VSV reduces eIF2α phosphorylation, promoting viral protein synthesis.	Increase in VSV replication.	In vitro	[124]
SARS-CoV-2	HIF-1α downregulates the expression of ACE2 and TMPRSS2 SARS-CoV-2 receptors.	Decrease of SARS-CoV-2 infection by a reduction of the release of viral particles in lung epithelial cells.	In vitro and in vivo in C57BL/6 mice	[125]
	HIF-1α induces Angiotensin II, which inhibits ACE2 synthesis.	Decrease of SARS-CoV-2 replication in human pulmonary artery smooth muscle cells.	In vitro	[126]

## Data Availability

Not applicable.
